# The efficacy and safety of dexmedetomidine in cardiac surgery patients: A systematic review and meta-analysis

**DOI:** 10.1371/journal.pone.0202620

**Published:** 2018-09-19

**Authors:** Guobin Wang, Jianhua Niu, Zhitao Li, Haifeng Lv, Hongliu Cai

**Affiliations:** Department of Surgical Intensive Care Unit, The First Affiliated Hospital, Medical College, Zhejiang University, Hangzhou, Zhejiang, P.R. China; University of Notre Dame Australia, AUSTRALIA

## Abstract

This study aimed to evaluate the efficacy and safety of dexmedetomidine versus any other treatment without dexmedetomidine in patients who have undergone cardiac surgery. Electronic databases including PubMed, Embase, and Cochrane Library were systematically searched without limitations of language and publication time. Randomized controlled trials (RCTs) aiming to evaluate the efficacy and safety of dexmedetomidine versus any other treatment without dexmedetomidine in patients that have undergone cardiac surgery were selected. Endpoints such as hemodynamic indexes and adverse events in eligible studies were extracted by two researchers, independently. The data was analyzed using RevMan 5.3 and Stata 11.0 software. A total of 18 RCTs met the inclusion criteria, involving 1730 patients. Compared to control (any treatment without dexmedetomidine), dexmedetomidine showed a pooled mean difference (MD) of -14.46 [95% confidence interval(CI): -24.69, -4.23; p<0.01] for systolic arterial pressure, a standardized mean difference (SMD) of -1.74 for mean arterial blood pressure (95% CI: -2.80, -0.68; P < 0.01), -2.12 (95%CI: -3.23, -1.00; p<0.01) for heart rate, and combined odds ratio (OR) of 0.22 (95%CI: 0.11, 0.44; p<0.01) for tachycardia, 3.44 (95%CI: 1.95, 5.96; p<0.01) for bradycardia, 0.74 (95%CI: 0.49, 1.12; p>0.05) for atrial fibrillation, and 0.99 (95%CI: 0.51, 1.90; p>0.05) for hypotension. In addition, dexmedetomidine could reduce time of surgery and stay in intensive care units, improve delirium with good safety. Our study shows clinical application of dexmedetomidine in cardiac surgery patients can reduce risks of abnormal hemodynamics with good safety.

## Introduction

More than 2 million cardiac surgeries are performed in the world annually [1]. While cardiac surgery is often used to treat complications of ischemic heart disease, correct congenital heart disease, or treat valvular heart disease from various causes, including endocarditis, rheumatic heart disease, and atherosclerosis, these procedures have several disadvantages [2–4]. Cardiac surgery is suggested to be associated with high risks of cardiovascular complications and other adverse events when performing operations, which are often resulted in increased hospital stay and even mortality [5–10]. Although great improvements in equipment, techniques and medical care have been achieved, and decreased the incidences of major complication rates and mortality [11–16], effective and safe perioperative medication is required to further reduce these negative events [8,11,17,18].

Dexmedetomidine is an anxiety reducing sedative, and pain medication [19]. It is notable for its ability to provide sedation without any risk of respiratory depression and can provide cooperative or semi-arousable sedation [20]. Several studies suggested that dexmedetomidine may be useful for the treatment of the negative cardiovascular effects of cardiac surgery [21–26]. However, the utilization of this medication was limited in clinical practice as its common side effects such as hypotension and bradycardia, and higher economic costs [11,27].

In this study, we explored the efficacy and safety of dexmedetomidine versus other medications in cardiac surgery patients. After systematic research, the effects of dexmedetomidine on hemodynamics in patients undergoing cardiac surgery compared with other drugs were noted. Patients undergoing cardiac surgery and even other drugs were found and selected. Data was analyzed by RevMan 5.3 and Stata 11.0 software to make evidence supporting the efficacy and safety of dexmedetomidine in patients undergoing cardiac surgery.

## Methods

This meta-analysis was performed by following the guidance of the Preferred Reporting Items for Systematic Reviews and Meta-Analyses (PRISMA)(S1 Checklist).

### Search strategy

Electronic databases such as PubMed, Embase, and the Cochrane Library were systematically searched for clinical RCTs comparing the efficacy and safety of dexmedetomidine versus other medications in treating patients undergoing cardiac surgery. The following Mesh terms and free texts were used in various combinations for selecting eligible studies: "dexmedetomidine", "cardiac surgery", "cardio protection", "myocardial", "cardiopulmonary bypass", "coronary artery bypass grafting ", "heart surgery", and "heart valve". Language restriction was not introduced in this study. Unpublished articles or outcomes were also searched by contacting relevant researchers of this topic. To move more towards a perfect search, the references of similar reviews, and major cardiac surgical scientific meetings’ abstracts were also selected for further evaluation. If necessary, manual retrieval of references would be applied.

### Inclusion and exclusion criteria

According to the participants, interventions, comparisons, outcomes, and study design (PICOS) protocol, the following criteria were used. Patients: who were diagnosed with complications of ischemic heart disease, or needed to treat congenital heart disease, valvular heart disease from various causes, including endocarditis, rheumatic heart disease, and atherosclerosis, and received cardiac surgery. Intervention and comparison: each study contained two comparison groups, one received dexmedetomidine, and the other group received control (treatment without dexmedetomidine). Outcomes: hemodynamics indexes including mean arterial pressure, mean pulmonary arterial pressure, pulmonary capillary wedge pressure, systemic vascular resistance index, pulmonary vascular resistance index, incidence of tachycardia/hypotension/bradycardia, heart rate and blood pressure. Other indicators such as pain score, satisfaction scores, and postoperative complications were also included. Study design: only randomized controlled trials were included to ensure the quality of pooled results. Retrospective study, observational study, reviews, and animal studies were excluded. If articles failed to provide sufficient information or data, they were also excluded.

### Data extraction

Two reviewers extracted the baseline characteristics and eligible endpoint data from included studies, independently. The basal information included first author, year of publication, number of patients in each group, mean age, sex, disease diagnostic criteria, cardiac surgery types, and application information of dexmedetomidine. Outcomes included mean systolic arterial pressure, mean arterial blood pressure, central venous pressure, mean pulmonary arterial pressure and heart rate, incidence of bradycardia or tachycardia, cardiac index, and duration of ICU and surgery. Other indicators such as postoperative complications were also extracted. If controversies about the recorded data existed, a third reviewer was introduced to solve it.

### Quality assessment

RCTs that met the inclusion criteria and passed the following assessment of quality were included for the final analysis. The quality assessment section of the Cochrane Handbook for Systematic Reviews of Interventions was used to evaluate the overall quality by two investigators, independently [28]. Quality items such as bias of selection, bias of blinding, bias of incomplete outcome data, bias of selective reporting, and other bias within each included study were assessed. If there were any disagreements, a third investigator was involved to solve the problem. According to the handbook mentioned above, the final quality was defined as low risk, moderate risk or high risk of bias, based on the results of the overall quality assessment.

### Statistical analysis

Meta-analysis was performed using the RevMan 5.3 software. To present the dichotomous data, the odds ratio (OR) with a 95% confidence interval (CI) was used to represent the effects of intervention on interest indicators over that of control. To present the continuous variables, the mean ± standard deviation was used. The mean difference (MD) was applied if the measurement unit of continuous variables was in accordance with each other. Otherwise the standardized mean difference (SMD) was used. The heterogeneity test was evaluated by Q test and I^2^ coefficient. In addition, the method for calculating standard deviation (SD), provided by the Handbook, was used to get indirect SD [28]. If the I^2^%> 75% suggested that there was obvious heterogeneity between the studies; if the I^2^%<40%, the study could be considered homogeneous; if the I^2^% was between 30% to 60%, a moderate heterogeneity was considered [28]. According to the results of the heterogeneity detection, the random effect model was used if there was significant heterogeneity; otherwise, the fixed effect model was used. When indicating significant heterogeneity, the subgroup analysis of the target data was introduced. The results of meta-analysis were presented in forms of forest plots. To detect publication bias, the funnel plot, Begg' test and Egger test was used. All significance testing was two-sided, and if P < 0.05 was considered as statistically significant.

## Results

### Search results

**Fig 1:**
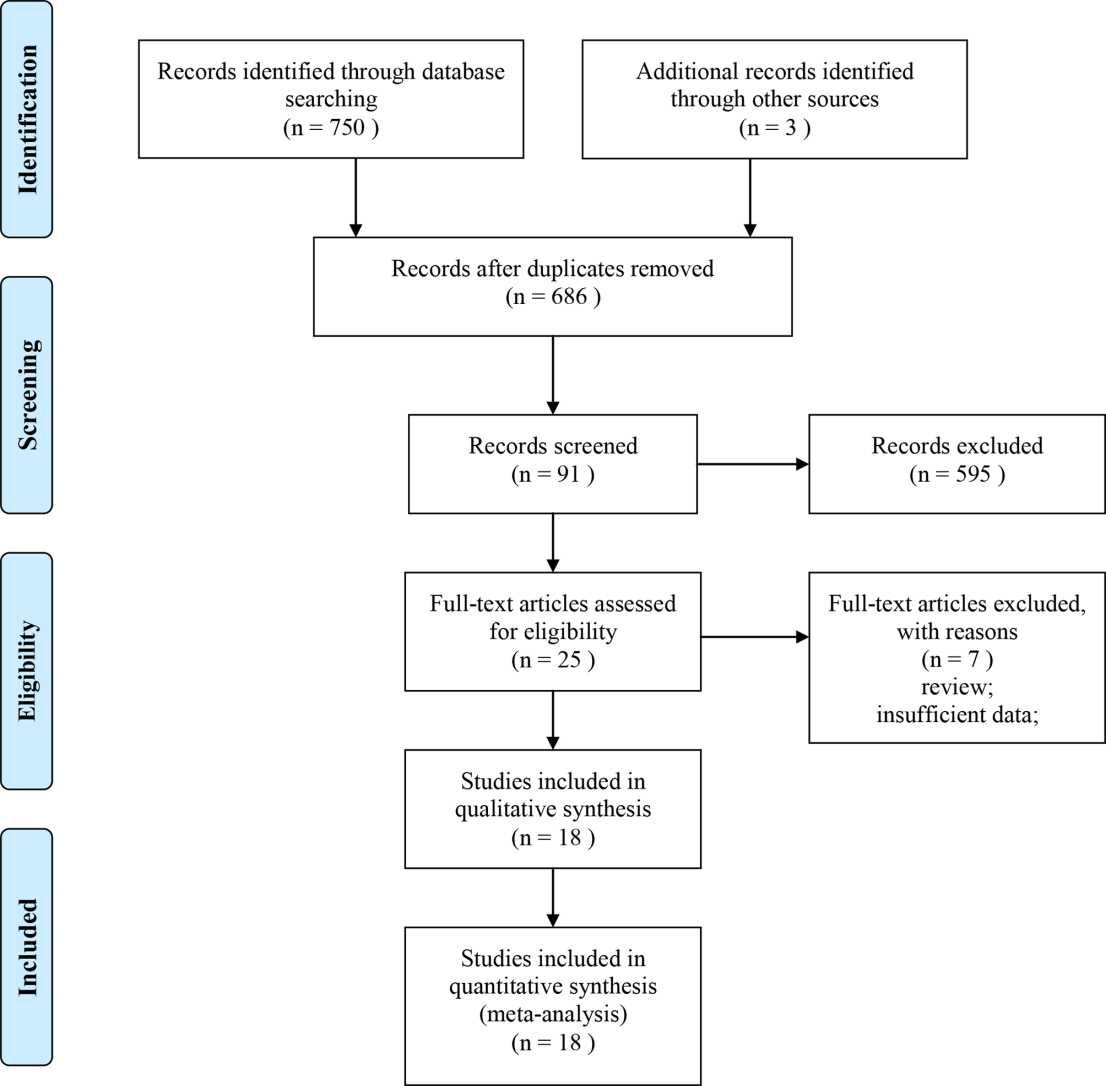
Flow diagram following the PRISMA guideline.

A total of 753 literatures were retrieved, and the literatures were screened strictly according to the inclusion and exclusion criteria by two reviewers, independently. 595 of these references were discarded after the first screen process. Further review of the abstract and full text of the remaining 91 articles was followed. At last, the final 18 RCTs [2,20–22,24,29–41] were included in this systematic review. Total of 1730 patients undergoing cardiac surgery were involved in these studies, including 865 cases in the dexmedetomidine group, and 865 cases in the control group (any treatment without dexmedetomidine). The baseline characteristics of the included studies are shown in Table 1. The detailed information of the study selection process is presented in **Fig 1**.

**Table 1:** Baseline characteristics of included studies.

First author	Year	Number of cases		Dex dose/administration	Surgery
		Dex	Con		
Herr	2003	148	147	1.0 μg/kg loading; 0.2 μg/kg/h infusion	Cardiac surgeries
Corbett	2005	43	46	1 μg/kg loading; 0.4 μg/kg continuous infusion	Coronary artery bypass grafting
Shehabi	2009	152	147	0.1 μg/kg/h	Cardiac surgeries
Rabie	2016	75	75	1μg/kg loading, maintained as an infusion of 0.3μg/kg/h	Cardiac surgeries
Ren	2013	81	81	0.2 μg/kg/h	Coronary artery bypass grafting
Tosun	2013	18	20	0.5 μg/kg loading; 0.5 μg/kg/min continuous infusion	Coronary artery bypass grafting
Aziz	2011	14	14	0.12 ± 0.03 ug/kg/h	CABG/septal repair/valvular repair
Balkanay	2015	30	30	8μg/cc	CABG with CPB
Jalonen	1997	40	40	50ng^/^kg^/^min for 30 min and followed by 7ng/kg^/^min	CABG with CPB
Maldonado	2009	40	38	loading dose:0.4ug/kg, followed by 0.2–0.7uk/kg/h	cardiac valve surgery
Chi	2016	34	33	1 μg/kg loading; 0.6 μg/kg continuous infusion	Off-pump coronary artery bypass grafting surgery
Liu	2016	29	32	1.5 μg/kg/h continuous infusion	Cardiac surgeries
Khalil	2016	25	25	1 μg/kg loading; 0.5 μg/kg/h continuous infusion	TAVI
Priye	2015	32	32	0.4μg/kg/h continuous infusion	elective cardiac surgery
Karaman	2015	31	33	0.2 μg/kg/h–1.0 μg/ kg/h	CABG with CPB
Eremenko	2014	28	27	0.2–0.7 μg/kg/h	cardiac surgery
Sulaiman	2012	30	30	0.5 μg/kg	cardiac surgery
Menda	2010	15	15	1 μg/kg	CABG

Abbreviation: Dex, dexmedetomidine; Con, control; M, male; F, female; NA, not available; CABG, coronary artery bypass grafting; TAVI, Transcatheter aortic valve implantation; CPB, cardiopulmonary bypass

### Quality of the included studies

**Fig 2:**
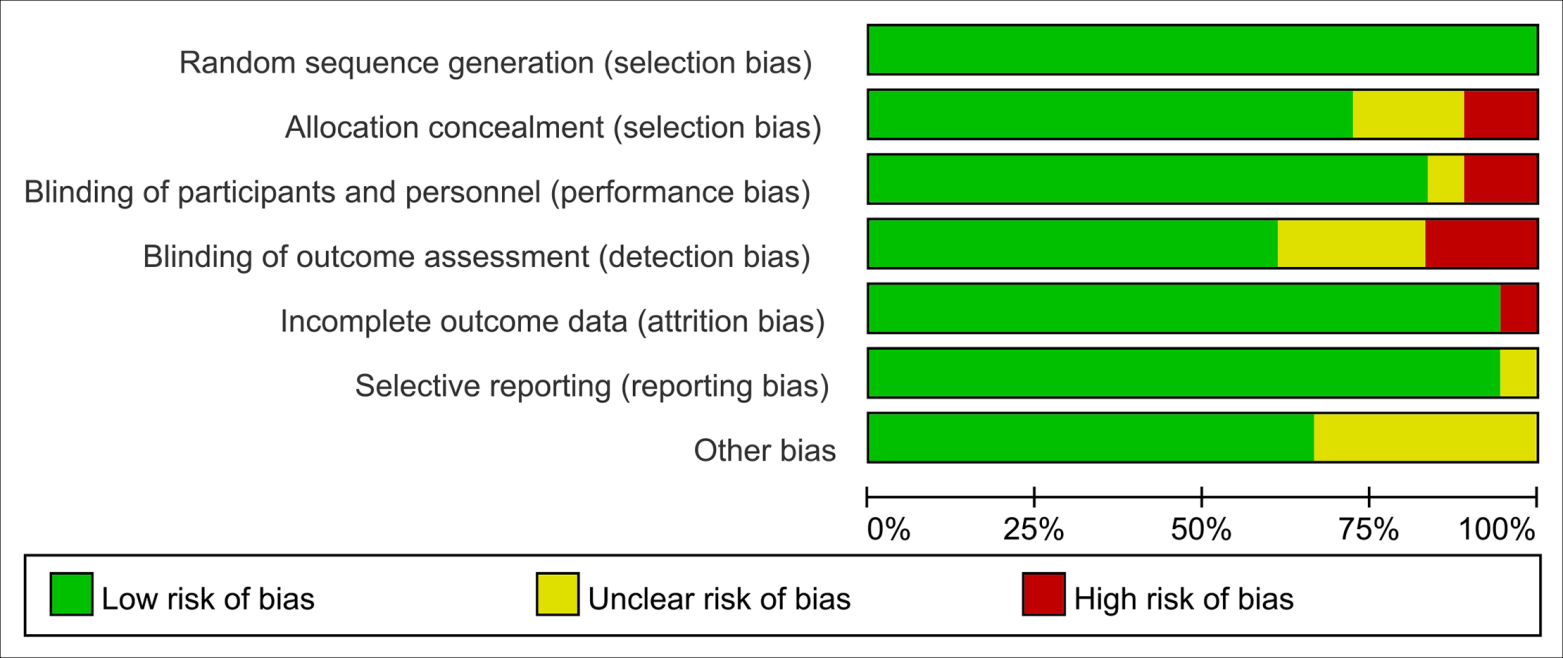
Risk of bias graph: Review authors' judgements about each risk of bias item presented as percentages across all included studies.

**Fig 3:**
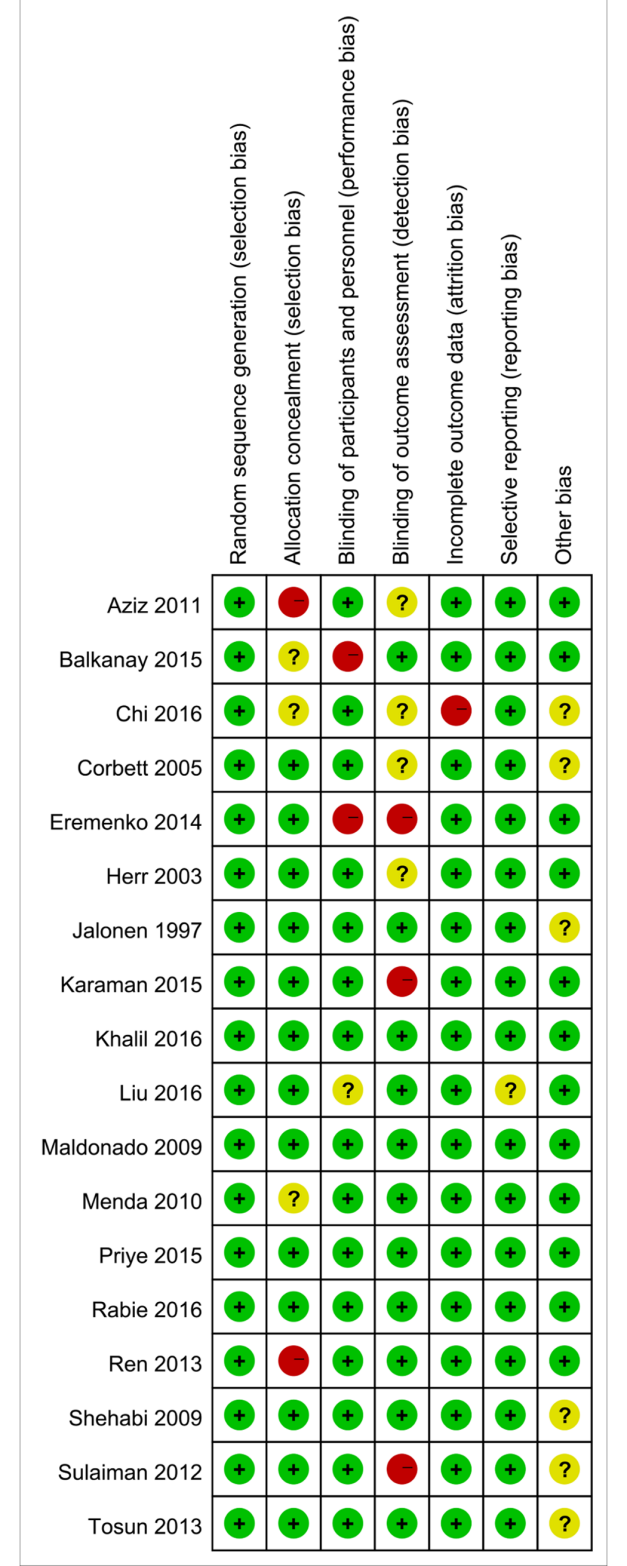
Risk of bias summary: Review authors' judgements about each risk of bias item for each included study.

Based on the quality assessment items of the Cochrane Handbook [28] for systematic review, all the included articles were evaluated for risk of biases, such as selection bias, selective reporting bias, incomplete reporting bias, and publication bias. The quality of the literatures was defined as low, moderate, and high risk of bias according to the Handbook mentioned above. Nearly all the included RCTs were evaluated as low risk of bias, indicating good qualities (**Figs 2 and 3**).

### Hemodynamic indexes

#### Systolic arterial pressure

**Fig 4:**
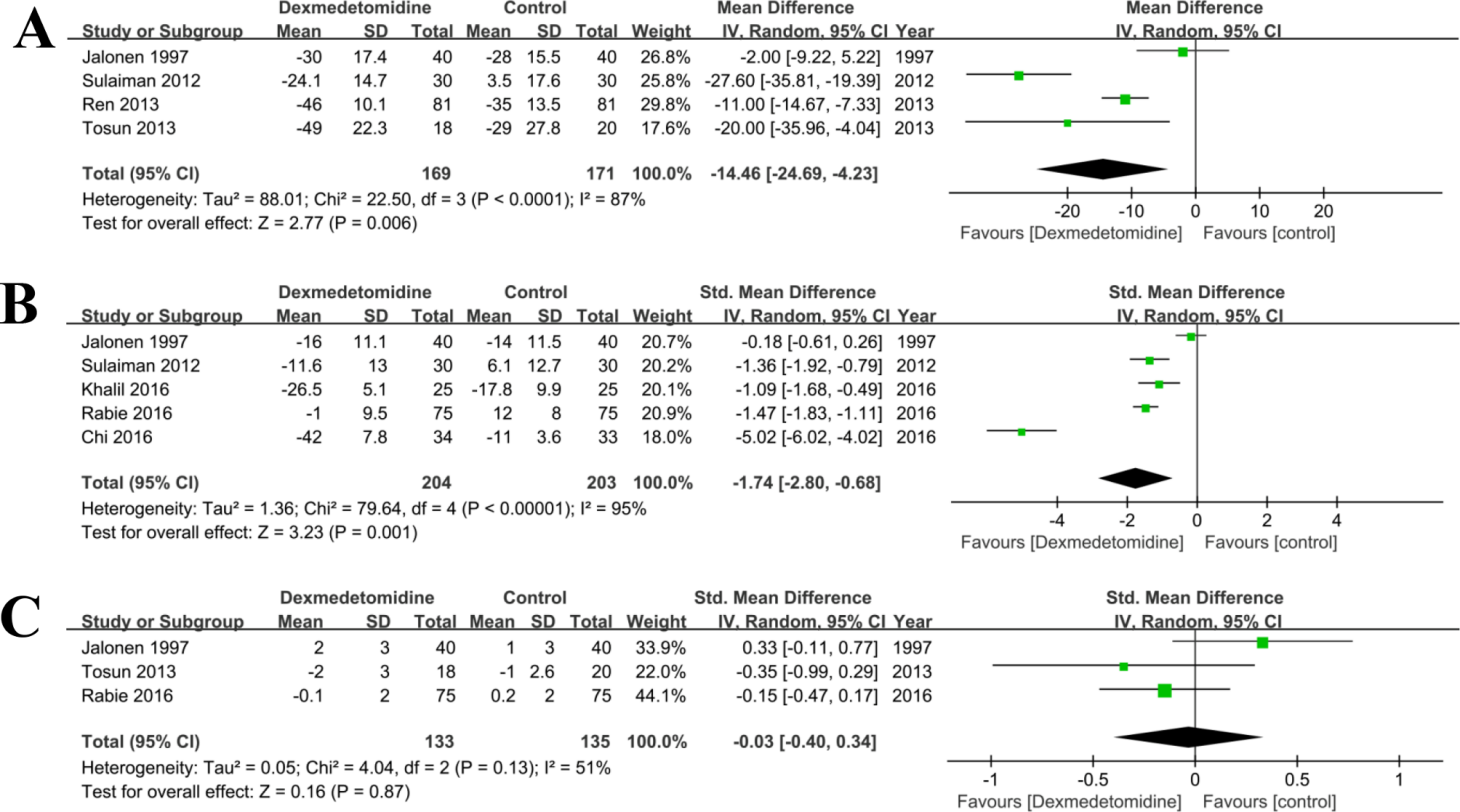
**Forest plot of comparison: 1 Dexmedetomidine VS. Control (any treatment without dexmedetomidine) for patients undergoing cardiac surgery, outcome**: A, Systolic arterial pressure; B, mean arterial blood pressure; C, Central venous pressure.

A meta-analysis of four studies [32–34,41] on dexmedetomidine versus control (any treatment without dexmedetomidine) found a significant difference in decreasing the systolic arterial pressure (MD = -14.46, 95% CI: -24.69, -4.23; P<0.0001, **Fig 4A**). As there was significant heterogeneity across these studies (I^2^% = 87%), the random effect model was used. The sensitivity analysis was used to detect the source of heterogeneity, and the result showed that the study of Tosun [32] might be responsible for it.

#### Mean arterial blood pressure

Five studies [2,20,22,34,41] provided data on mean arterial blood pressure. There was statistical heterogeneity among these studies (P<0.01, I^2^% = 95%). Combined results from the five RCTs of dexmedetomidine versus control (any treatment without dexmedetomidine) also found a significant difference in decreasing the mean arterial blood pressure (SMD = -1.74, 95% CI: -2.80, -0.68; P < 0.01, **Fig 4B**). The sensitivity analysis was used to detect the source of heterogeneity, and the result showed that the study of Chi [22] could be responsible for it.

#### Central venous pressure

There were three RCTs [2,32,41] compared the data of central venous pressure in patients undergoing cardiac surgery. There was a moderate heterogeneity among these studies (P = 0.13, I^2^ = 51%), and the random effect model was used. The combined results suggested that the central venous pressure in the dexmedetomidine group was similar to that in the control (any treatment without dexmedetomidine) group (SMD = -0.03, 95% CI: -0.40, 0.34; P = 0.87, **Fig 4C**). The sensitivity analysis was used to detect the source of heterogeneity, but did not discover the source.

#### Pulmonary artery mean pressure

**Fig 5:**
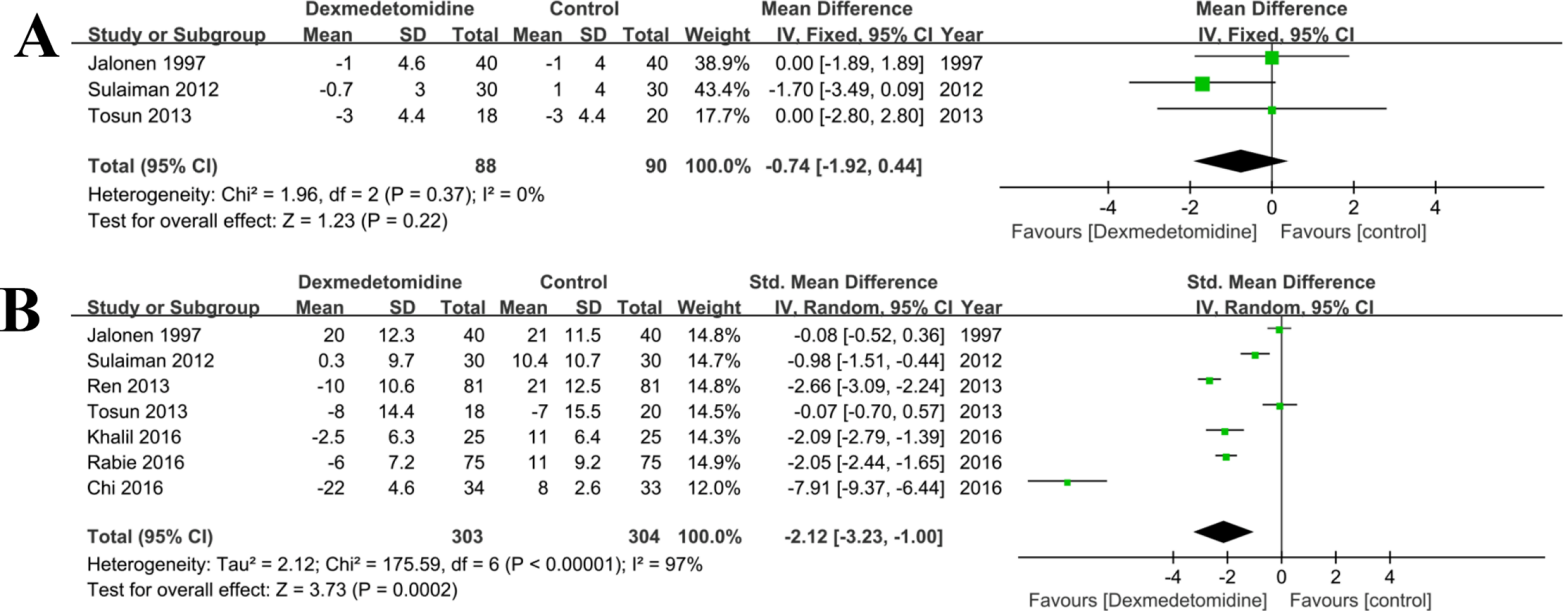
**Forest plot of comparison: 1 Dexmedetomidine VS. Control (any treatment without dexmedetomidine) for patients undergoing cardiac surgery, outcome**: A, Pulmonary artery mean pressure; B, Heart rate.

There were three RCTs [32,34,41] compared the data of pulmonary artery mean pressure in patients undergoing cardiac surgery. There was no statistical heterogeneity among these studies (P = 0.37, I^2^ = 0%), and the fixed effect model was used. The combined results suggested that the pulmonary artery mean pressure in the dexmedetomidine group was not significantly lower than that in the control (any treatment without dexmedetomidine) group (MD = -0.74, 95% CI: -1.92, 0.44; P = 0.22, **Fig 5A**).

### Other indicators

#### Heart rate

Seven RCTs [2,20,22,32–34,41] reported postoperative heart rates (**Fig 5B**). As there was significant heterogeneity (I^2^% = 97%), the random effect model was used. The results of meta-analysis showed that heart rates were lower in the dexmedetomidine group than in the control (any treatment without dexmedetomidine) group (MD = -2.12, 95% CI: -3.23, -1.00, P < 0.001). The sensitivity analysis was used to detect the source of heterogeneity, and the result showed that the study of Chi [22] could be responsible for it.

#### Bradycardia

**Fig 6:**
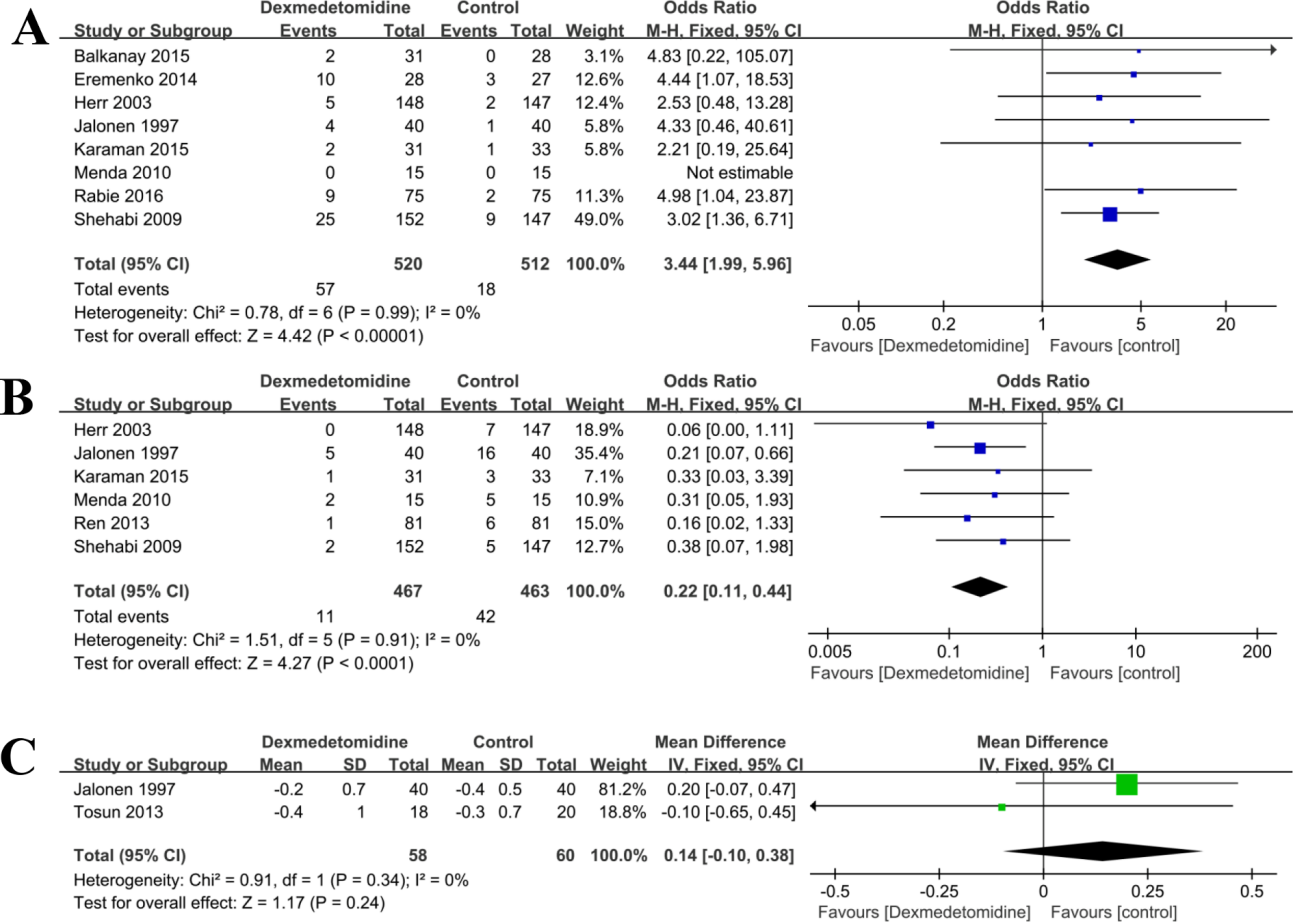
**Forest plot of comparison: 1 Dexmedetomidine VS. Control (any treatment without dexmedetomidine) for patients undergoing cardiac surgery, outcome**: A, Bradycardia; B, Tachycardia; C, Cardiac index.

There were eight studies [2,20–22,36,37,40,41] compared the incidences of bradycardia. There was no statistical heterogeneity (P = 0.99, I^2^ = 0%), and the fixed effect model was used. The results showed that significant difference in incidence of bradycardia was found between the dexmedetomidine group and the control (any treatment without dexmedetomidine) group (OR = 3.44, 95% CI: 1.99, 5.96; P<0.001, **Fig 6A**).

#### Tachycardia

Six studies [29,33,36,37,40,41] compared the rates of tachycardia of dexmedetomidine versus control (any treatment without dexmedetomidine) during surgery. There was no statistical heterogeneity (P = 0.91, I^2^ = 0%) in each study, and a fixed effect model was used. The results showed significant reduction in the prevalence of tachycardia in the dexmedetomidine group than that in the control (any treatment without dexmedetomidine) group (OR = 0.22, 95% CI: 0.11, 0.44; P <0.001, **Fig 6B**).

#### Cardiac index

Two studies [32,41] compared the cardiac index in the cardiac surgery perioperative period. There was no statistical heterogeneity (P = 0.34, I^2^ = 0%) in each study, and a fixed effect model was used. The results showed no significant difference between the two groups (OR = 0.14, 95% CI: -0.10, 0.38; P = 0.24, **Fig 6C**).

#### Duration of ICU stay and surgery

**Fig 7:**
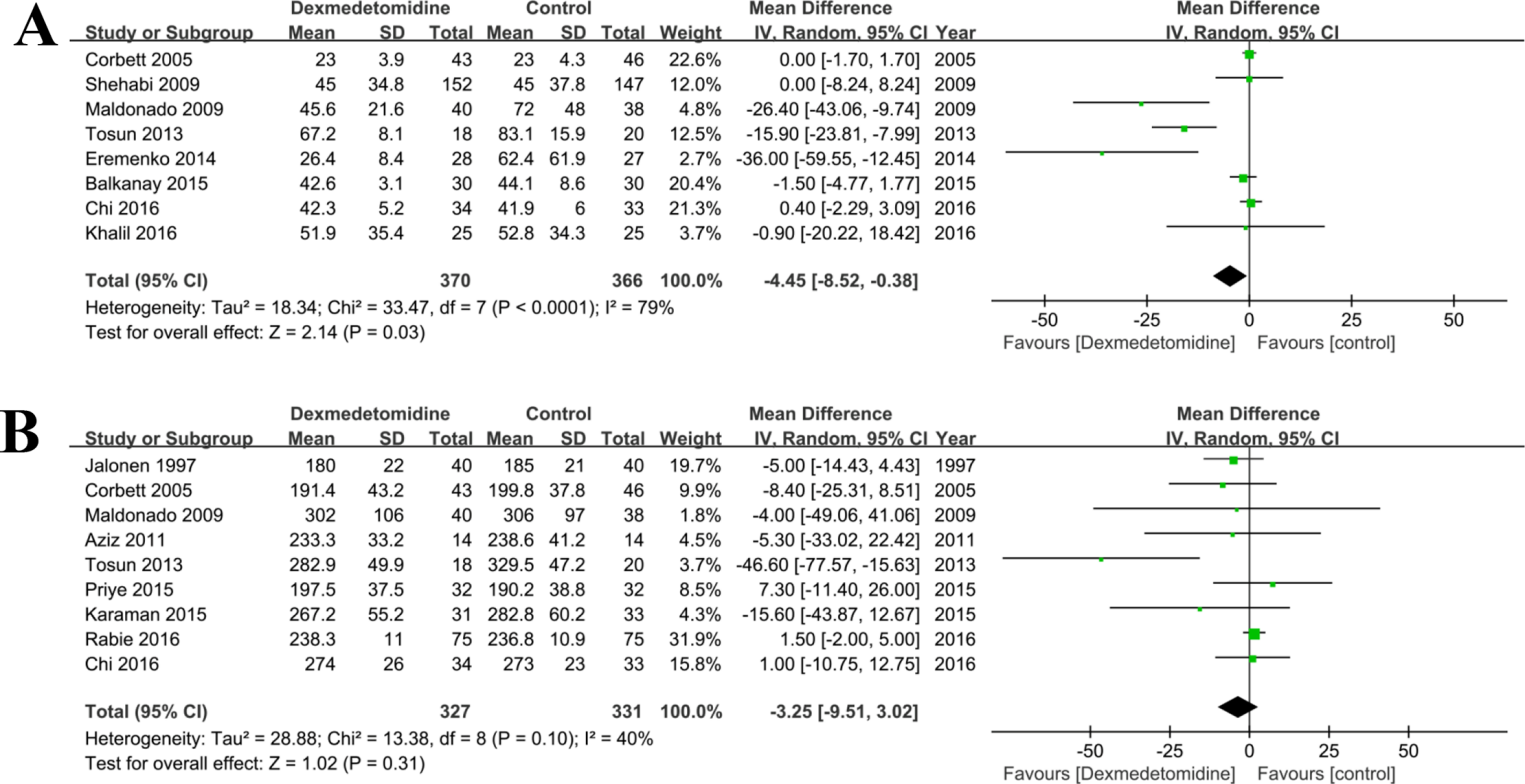
**Forest plot of comparison: 1 Dexmedetomidine VS. Control (any treatment without dexmedetomidine) for patients undergoing cardiac surgery, outcome**: A, ICU stay; B, Duration of surgery.

Eight [20,22,30–32,37–39] and nine [2,22,24,29,32,35,38,39,41] literatures compared the duration of ICU stay and surgery in patients treated with dexmedetomidine versus control group (any treatment without dexmedetomidine) during and after surgery (**Fig 7A and 7B**). There was significant heterogeneity among these studies (P<0.05), so the random effect model was introduced. Compared with the control group, the ICU stay (MD = -4.45, 95% CI: -8.52, -0.38; P = 0.03) was significantly decreased in dexmedetomidine than that in the control (any treatment without dexmedetomidine) group, but not the surgery time (MD = -3.25, 95% CI: -9.51, 3.02; P = 0.31).

### Postoperative complications

**Fig 8:**
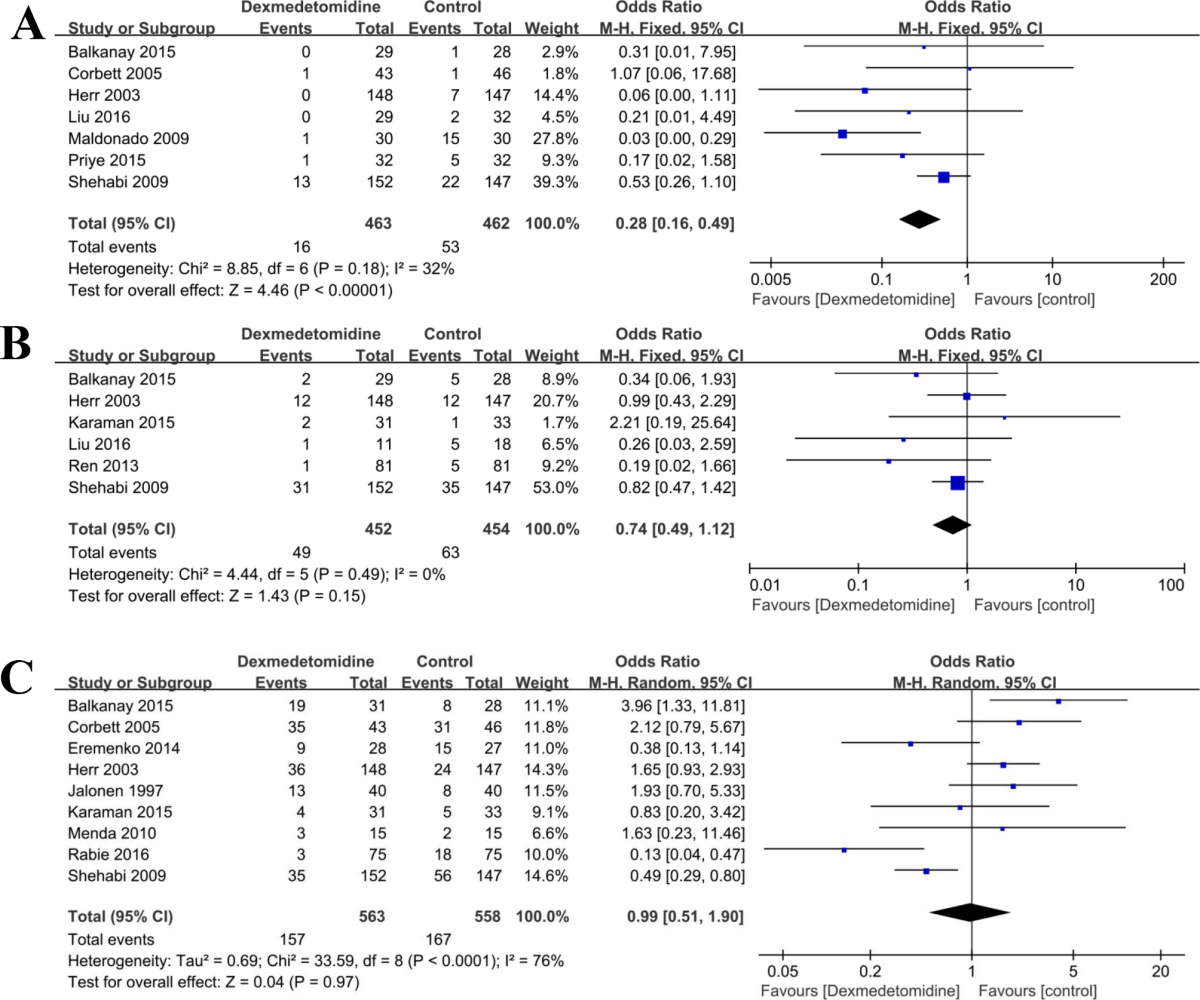
**Forest plot of comparison: 1 Dexmedetomidine VS. Control (any treatment without dexmedetomidine) for patients undergoing cardiac surgery, outcome**: A, Delirium; B, Atrial fibrillation; C, Hypotension.

The incidences of postoperative complications including delirium, atrial fibrillation, hypotension, and other adverse events such as renal failure, pulmonary edema, myocardial ischemia, and mortality of dexmedetomidine versus control (any treatment without dexmedetomidine) group in patients who received cardiac surgery were compared (Table 2). In brief, six studies [21,29,30,33,37,40] compared the incidence of atrial fibrillation in the cardiac surgery perioperative period. There was no statistical heterogeneity (P = 0.49, I^2^ = 0%) in any study, and a fixed effect model was used. The results showed no significant difference between the two groups (OR = 0.74, 95% CI: 0.49, 1.12; P = 0.15, **Fig 8B**).

**Table 2:** Summarized results of included studies.

Outcome	Studies	Participants	Statistical Method	Effect Estimate
Pulmonary artery mean pressure	3	178	Mean Difference (IV, Fixed, 95% CI)	-0.74 [-1.92, 0.44]
Heart rate	7	607	Mean Difference (IV, Random, 95% CI)	-15.22 [-23.50, -6.94]
Tachycardia	6	930	Odds Ratio (M-H, Fixed, 95% CI)	0.22 [0.11, 0.44]
Hypotension	9	1121	Odds Ratio (M-H, Random, 95% CI)	0.99 [0.51, 1.90]
Bradycardia	8	1032	Odds Ratio (M-H, Fixed, 95% CI)	3.44 [1.99, 5.96]
Central venous pressure	3	268	Mean Difference (IV, Random, 95% CI)	-0.06 [-1.02, 0.91]
Duration of surgery	9	658	Mean Difference (IV, Random, 95% CI)	-3.25 [-9.51, 3.02]
Mean arterial blood pressure	5	407	Mean Difference (IV, Random, 95% CI)	-14.54 [-25.09, -3.98]
Adverse events				
Renal failure	3	495	Odds Ratio (M-H, Fixed, 95% CI)	0.67 [0.28, 1.61]
Pulmonary edema	3	495	Odds Ratio (M-H, Fixed, 95% CI)	1.13 [0.43, 2.98]
Myocardial ischemia	4	657	Odds Ratio (M-H, Fixed, 95% CI)	0.42 [0.22, 0.80]
Mortality	4	588	Odds Ratio (M-H, Fixed, 95% CI)	0.66 [0.18, 2.35]
Delirium	6	630	Odds Ratio (M-H, Fixed, 95% CI)	0.32 [0.18, 0.57]
ICU stay	8	736	Mean Difference (IV, Random, 95% CI)	-4.45 [-8.52, -0.38]
Cardiac index	2	118	Mean Difference (IV, Fixed, 95% CI)	0.14 [-0.10, 0.38]
Systolic arterial pressure	4	340	Mean Difference (IV, Random, 95% CI)	-14.46 [-24.69, -4.23]
Atrial fibrillation	6	906	Odds Ratio (M-H, Fixed, 95% CI)	0.74 [0.49, 1.12]

Abbreviation: CI, confidence interval; ICU, intensive care unit.

**Fig 9:**
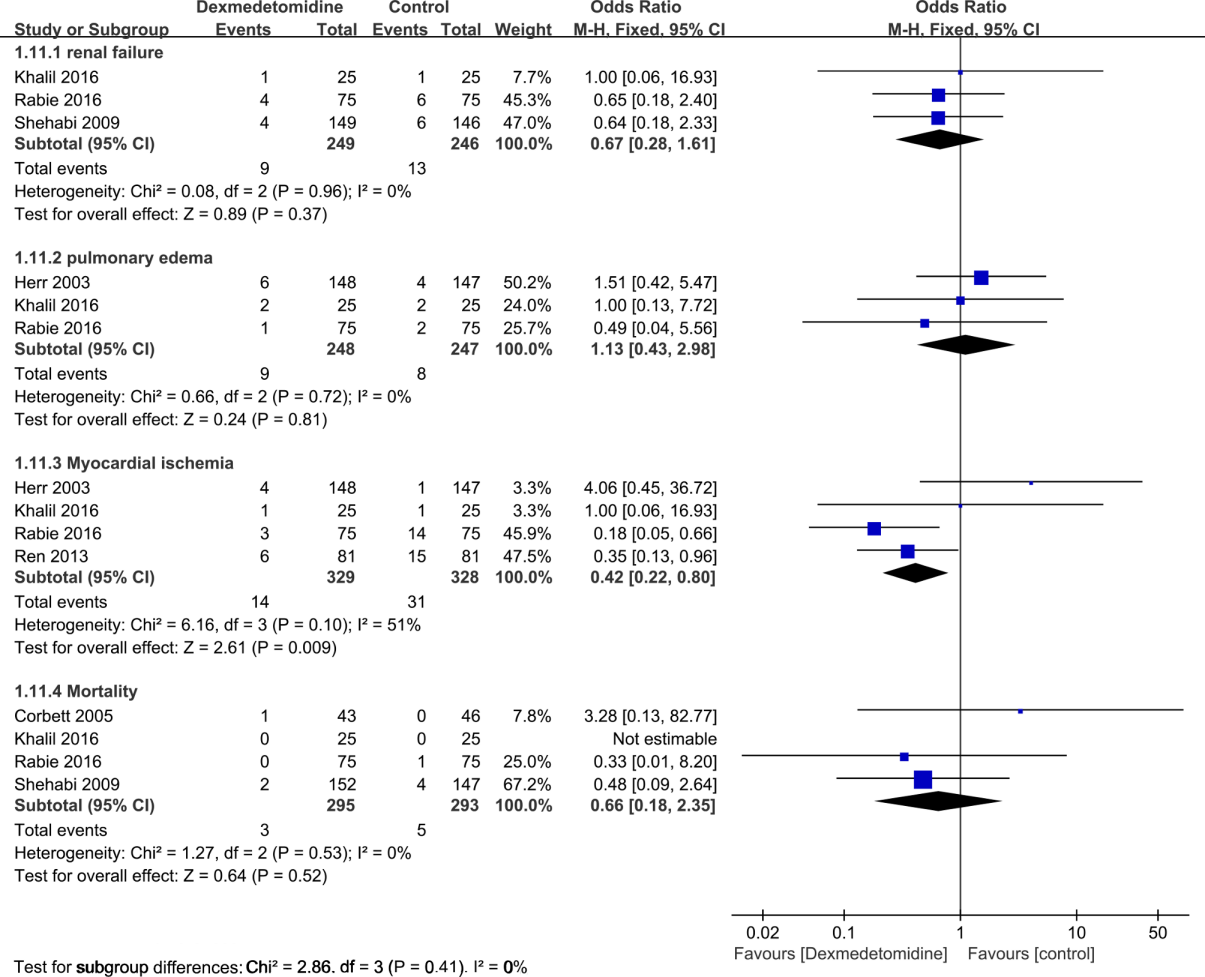
Forest plot of comparison: 1 Dexmedetomidine VS. Control (any treatment without dexmedetomidine) for patients undergoing cardiac surgery, outcome: Adverse events: renal failure; pulmonary edema; myocardial ischemia; mortality.

The hypotension (OR = 0.99, 95% CI: 0.51, 1.90; P = 0.97, **Fig 8C**), renal failure (OR = 0.67, 95% CI: 0.28, 1.61; P = 0.37, **Fig 9**), pulmonary edema (OR = 1.13, 95% CI: 0.43, 2.98; P = 0.81, **Fig 9**), and mortality (OR = 0.66, 95% CI: 0.18, 2.35; P = 0.52, **Fig 9**) were also similar between dexmedetomidine group and control group (any treatment without dexmedetomidine) (all p>0.05). However, the results showed that the incidences of delirium (OR = 0.32, 95% CI: 0.18, 0.57; P < 0.0001, **Fig 8A**), and myocardial ischemia (OR = 0.42, 95% CI: 0.22, 0.80; P = 0.009, **Fig 9**) were significantly lower when compared with control group (any treatment without dexmedetomidine) (**Table 2**).

### Publication bias assessment

Using the ICU stay as an endpoint, the possible publication bias was detected from the funnel plot (S1 Fig). As indicated by the Begg's test (p = 0.174) and Egger's test (p = 0.033), there may exist some bias. Then we followed the “trim and fill” method, and it showed no publication bias there.

The funnel plots of duration of surgery (S2 Fig), heart rate (S3 Fig), delirium (S4 Fig), atrial fibrillation (S5 Fig), bradycardia (S6 Fig), tachycardia (S7 Fig), and hypotension (S8 Fig) were also conducted to detect whether publication bias existed within these endpoints, and the results showed that there was no significant bias as indicated by the symmetry of the plots. Begg's tests (p = 0.348 for duration of surgery, p = 0.764 for heart rate, p = 0.764 for delirium, p = 1.000 for atrial fibrillation, p = 0.548 for bradycardia, p = 0.452 for tachycardia, p = 0.602 for hypotension) and Egger's tests (p = 0.084 for duration of surgery, p = 0.377 for heart rate, p = 0.335 for delirium, p = 0.843 for atrial fibrillation, p = 0.367 for bradycardia, p = 0.244 for tachycardia, p = 0.963 for hypotension) also proved no significant publication bias.

## Discussion

Although advancements in cardiac surgery have remarkably reduced the incidences of mortality and serious complications, effective medication is needed to benefit patients undergoing cardiac surgery. Several studies [19,37,42,43] have reported that dexmedetomidine may have beneficial effects on clinical outcomes in patients with cardiac surgery. However, strong supportive evidence is required. By pooling data from eligible RCTs, the use of dexmedetomidine led to significantly beneficial effects on systolic arterial pressure, mean arterial blood pressure, pulmonary artery mean pressure, and heart rate. We also observed that administration of dexmedetomidine was associated with a significant reduction in the duration of ICU stay and surgery, occurrence of postoperative delirium and the incidence of tachycardia. Though above results showed that dexmedetomidine had improved outcomes, it was associated with increased risk of bradycardia.

Dexmedetomidine is suggested to have sedative, anxiolytic, and analgesic abilities in patients undergoing cardiac surgery [17]. In our study, the meta-analysis suggested that patients that were treated with dexmedetomidine had a lower heart rate, along with lower mean arterial blood pressure, systolic arterial pressure, pulmonary artery mean pressure, and reduced ICU stay than those in the control group (any treatment without dexmedetomidine). The incidences of tachycardia, hypotension, and delirium decreased in the dexmedetomidine group, when compared to the control (any treatment without dexmedetomidine) group, indicating that patients who were treated with dexmedetomidine had lower risks of getting these events. Regarding bradycardia, the rate of this event was increased by about 3.4 times than those that did not use dexmedetomidine. This suggested that the heart rates of patients using dexmedetomidine should be carefully monitored. Other adverse events including renal failure, stroke, pulmonary edema, and mortality were also compared, and the results did not suggest that there were any significant differences between dexmedetomidine and other medications or placebo, proving the safety of dexmedetomidine.

Previously, a few meta-analyses and reviews [11,17,44,45], aiming to evaluate the effect of dexmedetomidine on clinical outcomes, delirium, myocardial protective, and postoperative complications were published. Lin et al. performed a meta-analysis [11] to assess whether dexmedetomidine could serve as a safe and efficacious sedative medication in post-cardiac surgery population. By analyzing the data, extracted from a total of 11 studies, the results of their meta-analysis showed that dexmedetomidine was associated with a shorter duration of mechanical ventilation, a lower prevalence of delirium, and tachycardia, but might increase the risk of bradycardia. They also found no significant difference in ICU stay between the different medications. With respect to postoperative complications, their results suggested that dexmedetomidine may not increase the risk of atrial fibrillation, hypotension, or mortality. Most of these results are in accordance with our findings, and we find a higher risk of bradycardia and a significant shorter duration of ICU stay. This may be explained by more included samples and cases of bradycardia, and decreased time of ICU stay in the included studies. More recently, another two meta-analyses [44,45] evaluated the myocardial protective effects and possibility of decreasing risks of delirium of dexmedetomidine in patients undergoing cardiac surgery or even post cardiac surgery. Gong et al. [44] included 18 studies and found that lower heart rate, systolic blood pressure, and incidence of tachycardia were associated with dexmedetomidine treatment in both adults and pediatric patients, and also elevated the number of bradycardia. In contrast, our meta-analysis included 18 studies and all of them were RCTs in adult patients. However, most of our findings were in accordance with their myocardial protective results in adult population. Liu et al. [45] included a total of 8 RCTs, and their results revealed that a lower risk of delirium, a shorter length of intubation, but a higher incidence of bradycardia were found in dexmedetomidine group as compared to propofol. There were no statistical differences in the risks of hypotension or atrial fibrillation, or the time of ICU stay between dexmedetomidine and propofol regimens. Unlike their ICU stay result, we found a significantly shorter stay in ICU.

All the included studies were RCTs, but potential heterogeneity may still exist within and across these trials, which may limit the quality of the results. Even though well designed and performed, there are still a few limitations in the present meta-analysis. First, the lack of gray literature (such as symposium records, unpublished data, government research reports, etc.) may lead to publication bias. Second, the qualities of the included RCTs were not in accordance with each other. Third, the time, dose of usage, injection speed of dexmedetomidine and disease and surgical type were different between the included studies, which may increase the heterogeneity bias across the studies and affect the results of the analysis. This suggests that the use of dexmedetomidine is not strictly organized. When different endpoints were set or goals were adopted, it was difficult to maintain consistency among various studies, which resulted in the apparent heterogeneity. We highlight the requirement for reliable and valid pooled results to determine the ideal efficacy of dexmedetomidine. Thus, we introduce heterogeneity detection method to minimize the potential influence of inconsistency to achieve the requirement mentioned above. There may be detection bias or measurement bias in the selection and evaluation of the cognitive function evaluation scale or the blood pressure, and the reliability is limited.

## Conclusion

In summary, the application of dexmedetomidine can effectively reduce the incidence of early postoperative delirium and ventricular tachycardia in patients who have undergone cardiac surgery, with tolerable adverse events. Our findings also suggest that dexmedetomidine may not increase the incidence of hemodynamic complications. Though the above conclusions are recommended, high-quality, large sample, randomized controlled clinical trials are needed to verify the effects and safety of dexmedetomidine in cardiac surgery patients.

## Supporting information

S1 Checklist PRISMA 2009 checklist. (DOC)

S1 Fig Funnel plot of comparison: Dexmedetomidine VS. Control (any treatment without dexmedetomidine) for patients undergoing cardiac surgery about ICU stay. (TIF)

S2 Fig Funnel plot of comparison: Dexmedetomidine VS. Control (any treatment without dexmedetomidine) for patients undergoing cardiac surgery about surgery time. (TIF)

S3 Fig Funnel plot of comparison: Dexmedetomidine VS. Control (any treatment without dexmedetomidine) for patients undergoing cardiac surgery about heart rate. (TIF)

S4 Fig Funnel plot of comparison: Dexmedetomidine VS. Control (any treatment without dexmedetomidine) for patients undergoing cardiac surgery about delirium. (TIF)

S5 Fig Funnel plot of comparison: Dexmedetomidine VS. Control (any treatment without dexmedetomidine) for patients undergoing cardiac surgery about atrial fibrillation. (TIF)

S6 Fig Funnel plot of comparison: Dexmedetomidine VS. Control (any treatment without dexmedetomidine) for patients undergoing cardiac surgery about bradycardia. (TIF)

S7 Fig Funnel plot of comparison: Dexmedetomidine VS. Control (any treatment without dexmedetomidine) for patients undergoing cardiac surgery about tachycardia. (TIF)

S8 Fig Funnel plot of comparison: Dexmedetomidine VS. Control (any treatment without dexmedetomidine) for patients undergoing cardiac surgery about hypotension. (TIF)
