# Supplementary figures and images for: The efficacy and safety of dexmedetomidine in cardiac surgery patients: A systematic review and meta-analysis
Source: PLoS One. 2018 Sep 19;13(9):e0202620. doi: 10.1371/journal.pone.0202620 (PMC6145508; doi:10.1371/journal.pone.0202620)

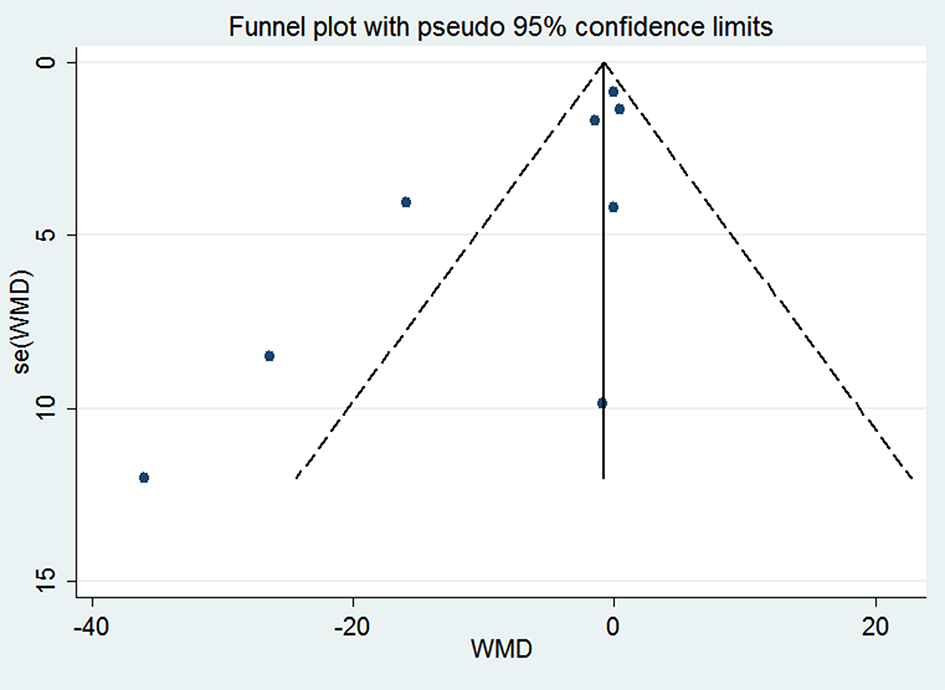

Supplement: S1 Fig — (TIF) [file pone.0202620.s002.tif]

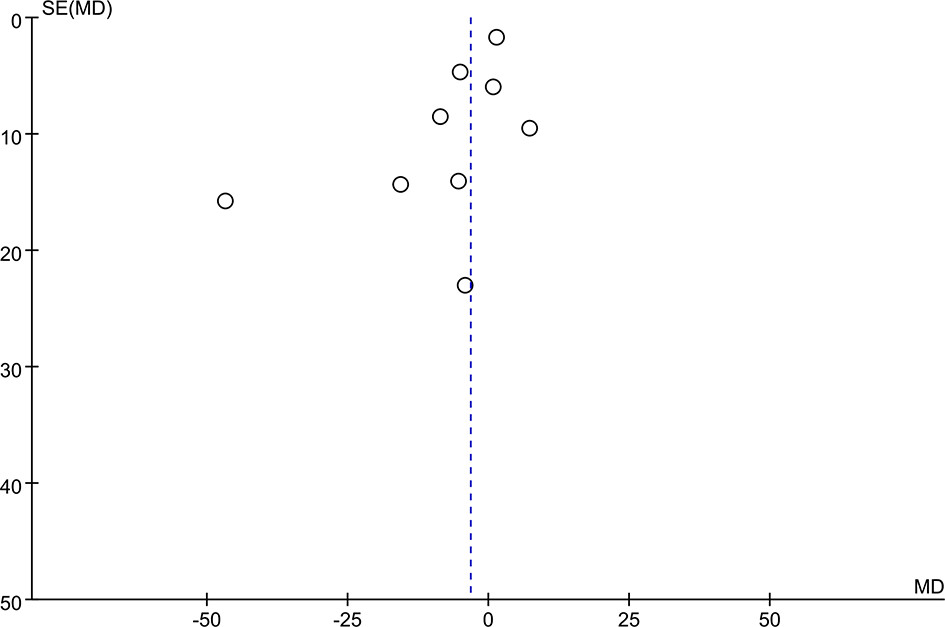

Supplement: S2 Fig — (TIF) [file pone.0202620.s003.tif]

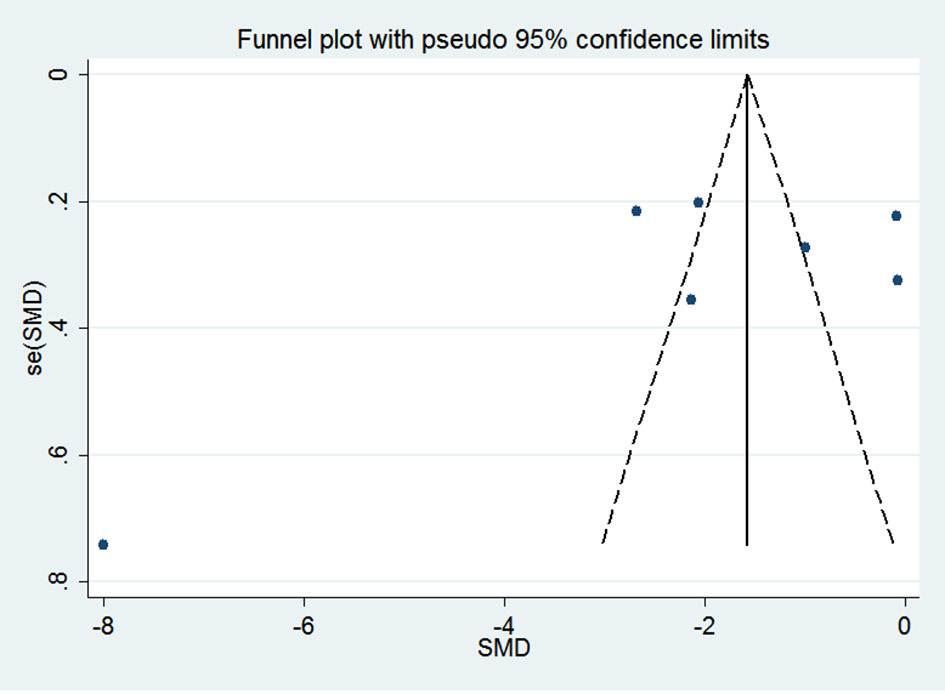

Supplement: S3 Fig — (TIF) [file pone.0202620.s004.tif]

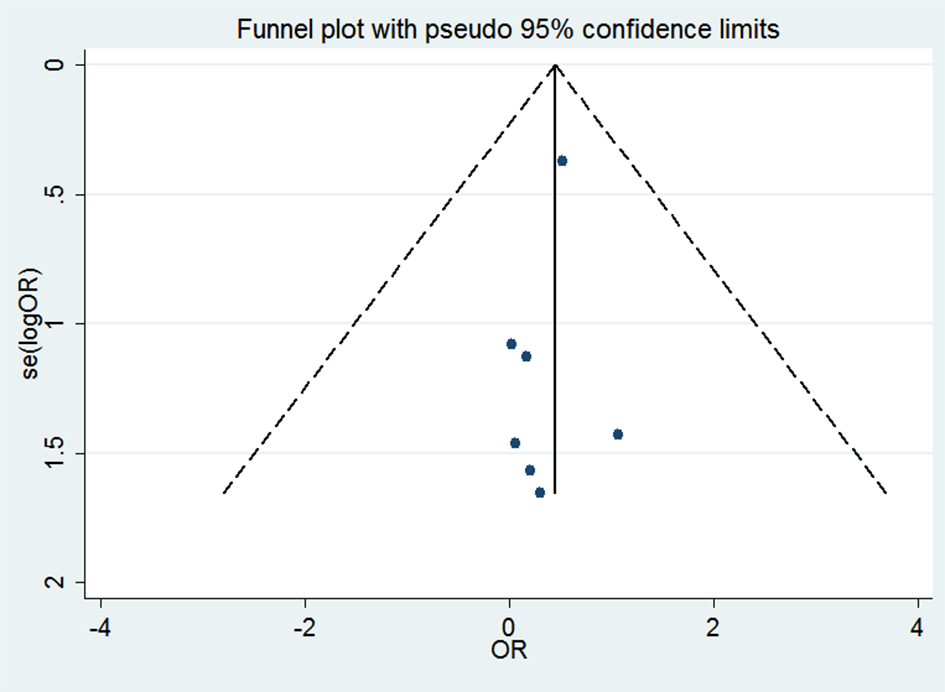

Supplement: S4 Fig — (TIF) [file pone.0202620.s005.tif]

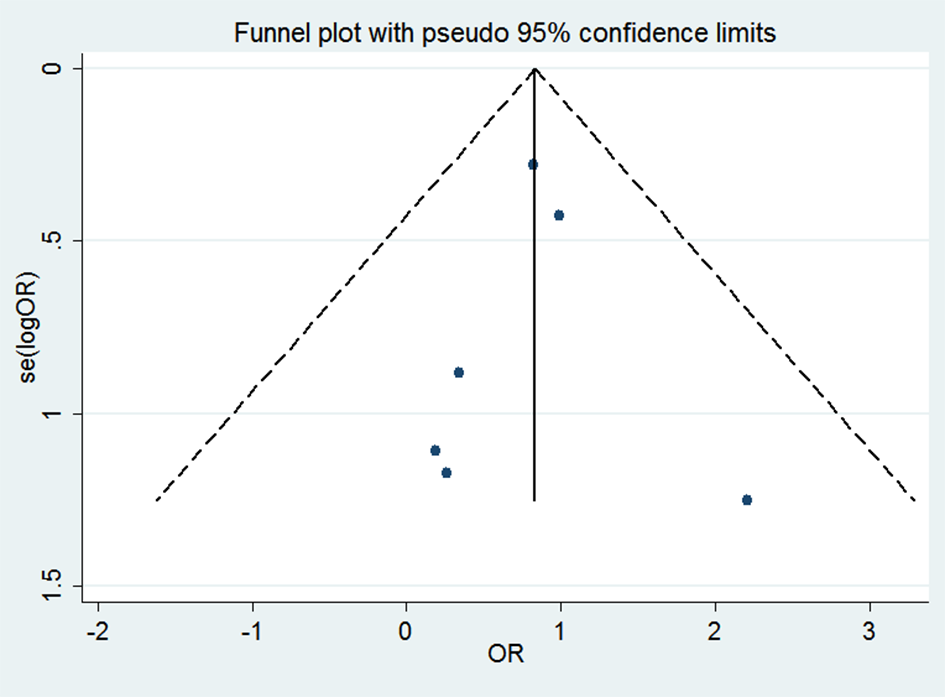

Supplement: S5 Fig — (TIF) [file pone.0202620.s006.tif]

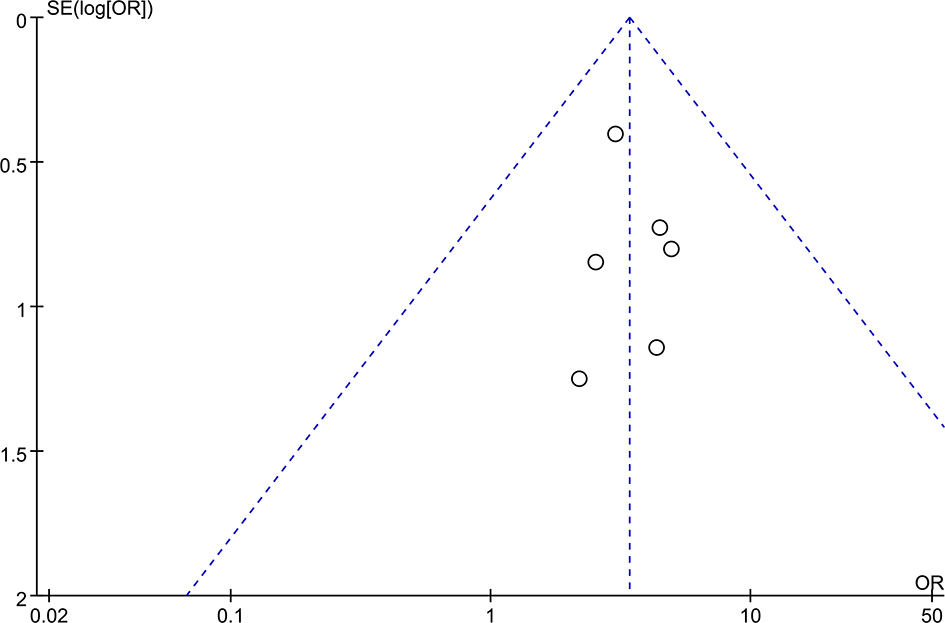

Supplement: S6 Fig — (TIF) [file pone.0202620.s007.tif]

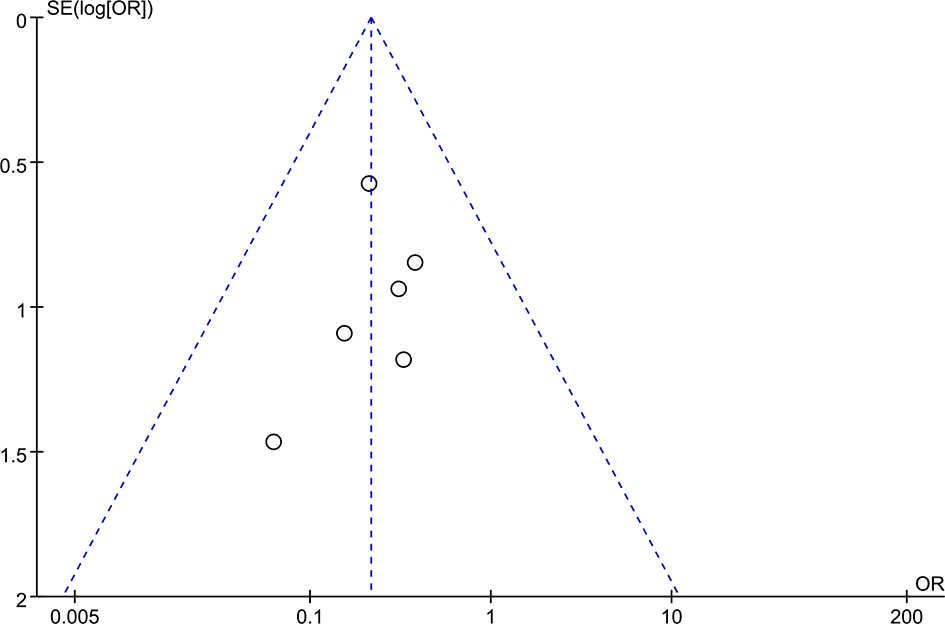

Supplement: S7 Fig — (TIF) [file pone.0202620.s008.tif]

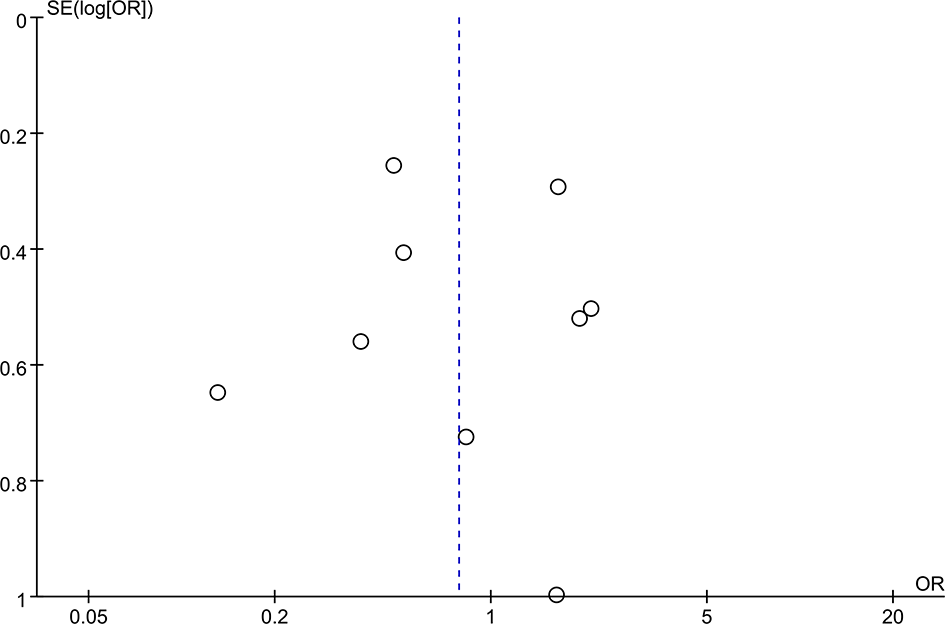

Supplement: S8 Fig — (TIF) [file pone.0202620.s009.tif]
